# Estimating Genetic Relatedness in Admixed Populations

**DOI:** 10.1534/g3.118.200485

**Published:** 2018-08-13

**Authors:** Arun Sethuraman

**Affiliations:** Department of Biological Sciences, California State University San Marcos, CA 92096

**Keywords:** Genetic Relatedness, Coancestry, Admixture, Population Structure

## Abstract

Estimating genetic relatedness, and inbreeding coefficients is important to the fields of quantitative genetics, conservation, genome-wide association studies (GWAS), and population genetics. Traditional estimators of genetic relatedness assume an underlying model of population structure. Each individual is assigned to a population, depending on *a priori* assumptions about geographical location of sampling, proximity, or genetic similarity. But often, this population assignment is unknown and assumptions about assignment can lead to erroneous estimates of genetic relatedness. I develop a generalized method of estimating relatedness in admixed populations, to account for (1) multi-allelic genomic data, (2) including all nine Identity By Descent (IBD) states, and implement a maximum likelihood based estimator of pairwise genetic relatedness in structured populations, part of the software, InRelate. Replicated estimations of genetic relatedness between admixed full sib (FS), half sib (HS), first cousin (FC), parent-offspring (PO) and unrelated (UR) dyads in simulated and empirical data from the HGDP-CEPH panel show considerably low bias and error while using InRelate, compared to several previously developed methods. I also propose a bootstrap scheme, and a series of Wald Tests to assign relatedness categories to pairs of individuals.

Estimating genetic relatedness is an important problem in biological statistics and population genetics. For instance, paternity or maternity assignment (see Avise 2001, Pearse *et al.* 2002, Yue and Chang 2010, Coleman and Jones 2011), and forensic studies (reviewed in Weir 2004) require a robust statistical framework to infer relatedness between genotyped individuals. Genetic relatedness also plays an important role in the study of quantitative traits where the proportion of trait variability explained by shared alleles indicates the strength of the genetic component of the trait (Falconer and Mackay 1996, Visscher *et al.* 2008). In several allied fields, accurate estimation of genetic relatedness is critical. For instance, association studies and linkage analyses without accounting for the increased relatedness due to population genetic structure could lead to spurious associations (Pritchard *et al.* 2000a). Genetic relatedness is also important in fields such as conservation genetics (Oliehoek *et al.* 2006, Wang 2018).

The genetic relatedness, $r_{XY},$ between two individuals *X* and *Y* can be defined in terms of the probability that their alleles are Identical By Descent (IBD). $r_{XY}$ is thus also twice the coefficient of coancestry, $\theta_{XY},$ and can be thought of as the inbreeding coefficient of any offspring that *X* and *Y* may sire (Weir *et al.* 2006).

Conventional relatedness estimators work in either of three ways: (1) estimating a coefficient of relatedness between two individuals using multilocus genotype data, and linkage data to inform the length of IBD tract sharing; or (2) assigning sib-ship partitions, reconstructing pedigrees, and using the pedigrees to estimate relatedness; or (3) directly estimating relatedness from known pedigrees (Weir *et al.* 2006). All relatedness estimators, however, have high variances, primarily owing to difficulty in parsing out true IBD states from observed Identity By State (IBS) states (Blouin 2003). This delineation of IBS *vs.* IBD is achieved by estimating the conditional probabilities of observing a genotype at a locus in one individual *X*, given the observed genotype at the same locus in individual *Y*.

In the presence of population genetic structure though, localized inbreeding makes individuals within the same subpopulation ‘more related’, than as suggested by their pedigree. Pervasive or specific inbreeding in recent generations past between two related individuals can be quantified though, if sufficient information is available on the existing genetic subpopulation structure. The estimated inbreeding coefficients (*e.g.*, *θ*, Weir 1994) affect the aforementioned conditional probabilities (Weir 1994). Alternately, maintenance of advantageous alleles in subpopulations by selection within a total population could also yield ‘artificial’ patterns of relatedness between individuals that share alleles, but not necessarily by direct descent.

Not accounting for such ‘shared’ allelic ancestry by utilizing true, or estimated subpopulation allele frequencies leads to incorrect estimates of genetic relatedness. Anderson and Weir (2007) subvert this issue of estimating subpopulation allele frequencies by directly quantifying the amount of inbreeding due to subpopulation structure, conditioned on *a priori* knowledge of existing subpopulations within a total population. Thus estimates of relatedness using the inbreeding coefficient *θ* in its formulation could be potentially biased.

Several other methods also utilize current population allele frequencies as proxies for ‘ancestral’ (this could mean subpopulation allele frequencies of the current generation, as in Anderson and Weir 2007, or allele frequencies of subpopulations from generations past, equated to current allele frequencies, as in Wang 2002) subpopulation allele frequencies, under Hardy-Weinberg Equilibrium (HWE), in their estimates of the inbreeding coefficient, *θ*. This assumption can be problematic because we do not know the precise number of ancestral subpopulations. However, the number of ancestral subpopulations can be approximated by the current subpopulation structure in a reference population.

Most methods for estimating pairwise genetic relatedness also assume that individuals whose pairwise relatedness is being estimated are derived from the same single, panmictic subpopulation. The methods of Anderson and Weir (2007), and Wang (2011b), that attempt to relax this assumption by handling samples from multiple subpopulations, assume that individuals derived from different subpopulations are genetically unrelated. However, in the presence of genetic admixture and migration, alleles are shared between subpopulations.

To account for unobserved population structure in bi-allelic genetic data, Moltke and Albrechtsen (2013) develop a two-step method (RelateADMIX), which estimates population genetic structure as admixture or ancestry proportions, and subpopulation allele frequencies. This method, when compared with other popular tools for estimating relatedness, including REAP (Thornton *et al.* 2012) and PLink (Purcell *et al.* 2007) shows considerable reduction in bias in estimating IBD probabilities. This method uses the following information: (a) admixture proportions of alleles at multiple bi-allelic loci in individuals, in “most likely” genetic subpopulations, as determined by likelihood or Bayesian methods such as those implemented in STRUCTURE (Falush *et al.* 2007), ADMIXTURE (Alexander *et al.* 2009), and MULTICLUST (Sethuraman 2013); and (b) subpopulation allele frequencies that are estimated as parameters in the model. Specifically, the model uses the probability distribution that an allele at a locus in an individual, or a multilocus genotype of an individual, was derived from a subpopulation in the recent past. IBS probabilities for two individuals, conditioned on the three IBD states $\left(D_{7},D_{8},D_{9}\right)$ *sensu* Jacquard 1972, Anderson and Weir 2007) are then calculated. This calculation contributes to a likelihood function (*sensu* Thompson 1975), which is then maximized using an Expectation Maximization (EM) algorithm (Dempster *et al.* 1977 to obtain maximum likelihood estimates for relatedness coefficients. These IBD coefficients are then used in calculating pairwise genetic relatedness, $r_{XY}$ and coancestry coefficients, $\theta_{XY}$ This method however assumes that alleles derived from different ancestral subpopulations are not IBD, and hence accounts for recent population structure. Here I develop an alternate formulation that utilizes estimated subpopulation allele frequencies, and ancestry proportions to estimate genetic relatedness in structured populations to include all nine IBD states $\left(D_{0-9}\right),$ and to be applicable to multi-allelic data, which accounts for ancestral subpopulation structure, where alleles derived from different subpopulations can also be IBD in an ancestral population. I develop a new package, InRelate based on a non-linear programming solution to this problem. I then address several questions based on the new framework 1) how does this estimator of pairwise genetic relatedness compare with other estimators of relatedness for structured and unstructured populations in simulated and empirical datasets?, 2) how does this estimator compare to other estimators with increase in available information (measured in terms of the number of genotyped loci)?, 3) how do bias and mean squared errors (MSE’s) in estimation using InRelate change with demographic model of evolutionary history?, 4) how does erroneous estimation of subpopulation structure due to label switching affect estimates of relatedness under the InRelate model? I also describe a method of bootstrapping and a series of statistical tests in order to obtain confidence intervals around estimates of relatedness.

## Materials and Methods

### Relatedness Under the Admixture Model

#### Theory:

I use the admixture model introduced by Pritchard *et al.* (2000b) to model population structure, since it makes few assumptions about the demography or history of the studied population.

It is to be noted that this model assumes that all individuals in the sample are unrelated, which in our case, is not actually true. If there are however, proportionately few relatives in the sample, then estimation under the admixture model should be reliable. For samples with rampant relatedness, pedigree estimation, or using methods that rely on linkage information may be more appropriate.

#### Data:

Assume that a sample of *I* largely unrelated, diploid individuals has been collected from a population possibly consisting of *K* unknown subpopulations. Each individual has been genotyped at *L* unlinked, codominant, neutrally evolving loci. Assume that locus *l* exhibits $A_{l}$ possible allelic states in the sample. For example, at SNP or AFLP presence/absence markers, $A_{l}=2.$ Microsatellite markers evolving under the infinite alleles model theoretically have infinite states, but we observe some $A_{l}<\infty$ in the finite sample. Missing data due to failed genotyping are allowed, but assumed to be missing completely at random.

The observed genotype data from diploids can then be combined into a three-dimensional matrix *X* of size I×L×2. Thus, $1\leq X_{ilm}\leq A_{l}$ is the *m*th (first or second) allele at a locus *l* in individual *i*. The data can then be reduced to sufficient statistics. Specifically, let $N=n_{ila}:1\leq i\leq I,1\leq l\leq L,1\leq a\leq A_{l}$ be a jagged array with entry $n_{ila},$ the number of alleles of type *a* observed at locus *l* in individual *i*.

### Relatedness Under the Admixture Model

The admixture model Pritchard *et al.* (2000b) posits that all the alleles in an individual are independent draws from a mixture of *K* subpopulations. Each subpopulation is characterized by its allele frequencies: $p_{kla}$ is the frequency of allele *a* $\left(1\leq a\leq A_{l}\right)$ at locus *l* (1≤l≤L) in subpopulation *k* (1≤k≤K). Each unrelated individual is characterized by a particular mixture of the *K* subpopulations: each allele of individual *i* (1≤i≤I) is derived from subpopulation *k* with probability $\eta_{ik}.$ The parameters are constrained such that $\sum_{k=1}^{K}\eta_{ik}=1$ for each individual *i*, and $\sum_{a=1}^{A_{l}}p_{kla}=1$ for each subpopulation *k* and locus *l*.

The likelihood of the observed multilocus genotype data, N, given the parameters $\Theta =\eta_{ik},p_{kla}:1\leq i\leq I,1\leq k\leq K,1\leq a\leq A_{l}$ under the admixture model is:

$$L\left(N|\text{Θ}\right)=\prod_{i=1}^{I}\prod_{l=1}^{L}\prod_{a=1}^{A_{l}}{\left(\sum_{k=1}^{K}\eta_{ik}p_{kla}\right)}^{n_{ila}}.$$ (1)

**Figure 1:**
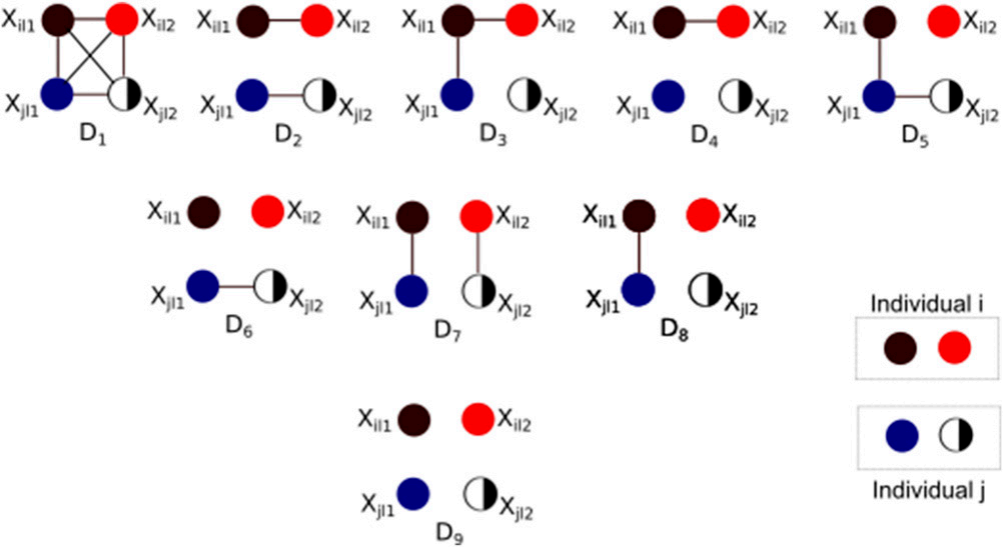
Nine possible Identity By Descent (IBD) states for the observed genotypes of two diploid individuals *i* and *j* at a genomic locus *l*. In each IBD state ($D_{1}-D_{9}$), The alleles are connected by a line if they are IBD. Observed Identity By State (IBS) states are not shown.

Relatives are then characterized by their shared alleles, *i.e.*, shared alleles that are identical by descent (IBD). As shown in Figure 1, the four alleles at a locus in two diploid individuals can be in one of nine possible, unobserved IBD states, $\text{Δ}=D_{1},D_{2},...,D_{9}.$ The marginal probability distribution over the IBD states for a pair of individuals at a locus is determined by their relationship. I use the notation $\delta_{q}=P\left(D_{q}\right)$ for this distribution. For example, in non-inbred populations, unrelated pairs are in state $D_{9}$ with probability $\delta_{9}=1,$ while full siblings will share no alleles at a locus with probability $\delta_{9}=0.25,$ one allele with probability $\delta_{8}=0.5,$ and both alleles with probability $\delta_{7}=0.25,$ assuming their parents are unrelated.

We only know if alleles are identical in state (IBS), and each IBD state is consistent with one or more of the nine IBS states, $S=S_{1},S_{2},...,S_{9}.$ Methods of relatedness estimation, use the IBS states observed at multiple, independent loci of two individuals to estimate *δ*, and hence their relationship.

Consider two individuals *i* and *j*. We observe their IBS state, $Y_{l}=X_{il1},X_{il2},X_{jl1},X_{jl2},$ at each locus *l*, where $a_{1}$ Each $Y_{l}$ follows an observed configuration in S, but the true IBD state, $Z_{l},$ is unobserved. Given a known relationship, R, between *i* and *j*, the likelihood of the observed data are

$$P\left(Y|R\right)=\prod_{l=1}^{L}P\left(Y_{l}|R\right)=\prod_{l=1}^{L}\sum_{s}^{1,2,\ldots ,9}P\left(Y_{l}|Z_{l}=D_{s},R\right)P\left(D_{s}|R\right)$$ (2)

If two individuals were full siblings from parents from the same subpopulation, genetic relatedness estimated using ancestral subpopulation frequencies would be expected to account for deep descent, and potential inbreeding of the parents. The relatedness between these full siblings, estimated using the parameters of the admixture model, should be as close to the true estimate, *i.e.*, $r_{XY}=0.5,$ as possible. On the other hand, if two individuals are full siblings from parents derived from two different subpopulations, genetic relatedness estimated using current subpopulation allele frequencies would likely be an over- or under-estimate, because the recent admixture event between the two parents in the previous generation is not accommodated. This result permits defining conditional probabilities of IBS states, given their IBD state using this new parametrization, *sensu* Jacquard (1972).

Following the leads of Jacquard (1972), Anderson and Weir (2007), and Wang (2011b), define the set of nine IBD states (see Figure 1), $\left\{D_{1},D_{2},...,D_{9}\right\}$ given a diploid locus between two individuals, 1 and 2. Each IBD state could have nine, or more possible IBS states, $\left\{S_{1}S_{2},...,S_{9}\right\}.$. Under the above assumptions, the probability that an allele $a_{p},$ is observed at a locus *l*, in individual *i* is $\sum_{k=1}^{K}p_{kla_{p}}\eta_{ik}=Z_{pi},$ the probability that an allele $a_{q},$ observed at the same locus *l*, in individual *j* is $\sum_{k=1}^{K}p_{kla_{q}}\eta_{jk}=Z_{qj},$ and so on. All the conditional probabilities, $P\left(S_{x}|D_{y}\right)$ are shown in Table 1. The likelihood of the IBD states over a single locus, L(X|Δ) can be written as

**Table 1:** Conditional Probabilities $P\left(S_{p}|D_{q}\right)$

Identity By Descent Mode										
IBS Mode	Allelic State	$D_{1}$	$D_{2}$	$D_{3}$	$D_{4}$	$D_{5}$	$D_{6}$	$D_{7}$	$D_{8}$	$D_{9}$
$S_{1}$	$A_{i}A_{i},A_{i}A_{i}$	$\frac{Z_{i1}+Z_{i2}}{2}$	$Z_{i1}Z_{i2}$	$Z_{i1}Z_{i2}$	$\frac{Z_{i1}Z_{i2}^{2}+Z_{i1}^{2}Z_{i2}}{2}$	$Z_{i1}Z_{i2}$	$\frac{Z_{i1}Z_{i2}^{2}+Z_{i1}^{2}Z_{i2}}{2}$	$Z_{i1}Z_{i2}$	$\frac{Z_{i1}^{2}Z_{i2}+Z_{i1}^{2}Z_{i1}}{2}$	$Z_{i1}^{2}Z_{i2}^{2}$
$S_{2}$	$A_{i}A_{i},A_{j}A_{j}$	0	$\frac{Z_{i1}Z_{j2}+Z_{j1}Z_{i2}}{2}$	0	$\frac{Z_{i1}Z_{j2}^{2}+Z_{j1}Z_{i2}^{2}}{2}$	0	$\frac{Z_{i1}Z_{j2}^{2}+Z_{j1}Z_{i2}^{2}}{2}$	0	0	$\frac{Z_{i1}^{2}Z_{j2}^{2}+Z_{j1}^{2}Z_{i2}^{2}}{2}$
$S_{3}$	$A_{i}A_{i},A_{i}A_{j}$	0	0	$\frac{Z_{i1}Z_{j2}+Z_{j1}Z_{i2}}{2}$	$\frac{Z_{i1}Z_{i2}Z_{j2}+Z_{j1}Z_{i2}Z_{j2}}{2}$	0	0	0	$\frac{Z_{i1}Z_{i2}Z_{j2}+Z_{j1}Z_{i2}Z_{j2}}{2}$	$\frac{Z_{i1}^{2}Z_{i2}Z_{j2}+Z_{j1}^{2}Z_{i2}Z_{j2}}{2}$
$S_{4}$	$A_{i}A_{i},A_{j}A_{k}$	0	0	0	$\frac{Z_{i1}Z_{j2}Z_{k2}+Z_{i2}Z_{j1}Z_{k1}}{2}$	0	0	0	0	$\frac{Z_{i1}^{2}Z_{j2}Z_{k2}+Z_{i2}^{2}Z_{j1}Z_{k1}}{2}$
$S_{5}$	$A_{i}A_{j},A_{i}A_{i}$	0	0	0	0	$\frac{Z_{i1}Z_{j2}+Z_{j1}Z_{i2}}{2}$	$\frac{Z_{i1}Z_{i2}Z_{j2}+Z_{j1}Z_{i2}Z_{j2}}{2}$	0	$\frac{Z_{i1}Z_{i2}Z_{j2}+Z_{j1}Z_{i2}Z_{j2}}{2}$	$\frac{Z_{i1}^{2}Z_{i2}Z_{j2}+Z_{j1}^{2}Z_{i2}Z_{j2}}{2}$
$S_{6}$	$A_{j}A_{k},A_{i}A_{i}$	0	0	0	0	0	$\frac{Z_{i1}Z_{j2}Z_{k2}+Z_{i2}Z_{j1}Z_{k1}}{2}$	0	0$\frac{Z_{i1}^{2}Z_{j2}Z_{k2}+Z_{i2}^{2}Z_{j1}Z_{k1}}{2}$	
$S_{7}$	$A_{i}A_{j},A_{i}A_{j}$	0	0	0	0	0	0	$\frac{Z_{i1}Z_{j2}+Z_{j1}Z_{i2}}{2}$	$0.5*\left\{Z_{j1}Z_{j2}\frac{Z_{i1}+Z_{i2}}{2}+Z_{i1}Z_{i2}\frac{Z_{j1}+Z_{j2}}{2}\right\}$	$Z_{i1}Z_{i2}Z_{j1}Z_{j2}$
$S_{8}$	$A_{i}A_{j},A_{i}A_{k}$	0	0	0	0	0	0	0	$\begin{array}{c} 0.5\text{*}\left\{\frac{Z_{i1}+Z_{i2}}{2}\left(Z_{j1}Z_{k2}+Z_{k1}Z_{j2}\right)\right\} \end{array}$	$0.5*\left\{Z_{i1}Z_{i2}\left(Z_{j1}Z_{k2}+Z_{j2}Z_{k1}\right)\right\}$
$S_{9}$	$A_{i}A_{j},A_{k}A_{l}$	0	0	0	0	0	0	0	0	$\frac{Z_{i1}Z_{j1}Z_{k2}Z_{l2}+Z_{k1}Z_{l1}Z_{i2}Z_{j2}}{2}$

$$L\left(X|\Delta \right)=P\left(S_{x}|\Delta \right)=\sum_{y}^{1,2,\ldots ,9}P\left(S_{x}|D_{y}\right)\Delta_{y}$$ (3),

where Δ is the set of 9 IBD states observable, *X* is the observed data, and $S_{x}$ is the observed IBS state of x∈X. Over *L* independent loci, this likelihood can be written as a product of individual locus likelihoods as

$$L\left(X|\Delta \right)=\prod_{l}^{L}P\left(S_{x}|\Delta \right)=\prod_{l}^{L}\sum_{y}^{1,2,\ldots ,9}P\left(S_{x}|D_{y}\right)\Delta_{y}$$ (4)

This likelihood function can be maximized using the constraints that each IBD coefficient, $\Delta_{y},$ y∈1,...,9 is ≥0 and ≤1, and $\sum_{y}^{1,\ldots ,9}\Delta_{y}=1.$ I used the solnp function in the Rsolnp package in *R* (Ghalanos and Theussl 2012), which implements the augmented Lagrange method of Ye (1988) to solve this nine-dimensional problem with linear constraints. The coancestry coefficient, $\theta_{XY},$ between two individuals, *X* and *Y* then can be calculated as $\theta_{XY}=\Delta_{1}+\frac{1}{2}\left(\Delta_{3}+\Delta_{5}+\Delta_{7}\right)+\frac{1}{4}\Delta_{8}$ and, by definition, the relatedness as $r_{XY}=2\theta_{XY}.$ Note that $r_{XY}$ is ≤1 only if the population is outbred ($\Delta_{j},j=1,...,6=0,$ and $\Delta_{7},\Delta_{8},\Delta_{9}\neq 0$).

### Other Relatedness Estimators

I also implemented the methods of Anderson and Weir (2007), and Wang (2011b) under the same optimization framework, using Rsolnp. The method of Wang (2011b) is different from that of Anderson and Weir (2007) in that it accounts for inbreeding. In both cases, subpopulation allele frequencies are modeled under the Dirichlet distribution, with the global parameter, *θ*, measured as the probability that two randomly sampled individuals from a subpopulation are IBD under an island model. Anderson and Weir (2007) do not state explicitly how they estimate *θ*, but Wang (2011b) indicates using the Weir and Cockerham *θ* estimator Weir and Cockerham (1984), which I used as well in the framework of Anderson and Weir (2007) (and Wang 2011b) to obtain comparable relatedness estimates. Regardless, under the equilibrium assumption that population subdivision is unchanging in time, the probability of drawing two of the same *a* alleles at a locus from the same subpopulation is $p_{a}+\left(1-\theta \right)p_{a},$ where $p_{a}$ is the frequency of allele *a* at that locus. This leads into the same likelihood framework described above (3,4), for the estimators of Anderson and Weir (2007), and Wang (2011b). Anderson and Weir (2007) utilize a simplex method to obtain maximum likelihood estimates of the IBD coefficients, $\Delta_{7},$ $\Delta_{8}$ and $\Delta_{9},$ using the constraints that $\sum_{i=7}^{9}\Delta =1,$ $0\leq \Delta_{i}\leq 1,$ and $4\Delta_{7}\Delta_{9}<\Delta_{8}^{2},$ for large, non-inbred populations.

Wang (2011b) offers another numerical solution by using Powell’s quadratically convergent method (Press 2007) to obtain likelihood estimates for all 9 variables above, as well as derives moment estimators under the same population structure framework, accounting for inbreeding using the inbreeding coefficient, *θ*, for other previously derived estimators (Queller and Goodnight 1989, Lynch and Ritland 1999, Wang 2002).

In this manuscript, the same non-linear programming method in 9 variables ($\Delta_{i},i\in 1,2,...,9$) was used to obtain maximum likelihood estimates for both estimators of Anderson and Weir (2007) and Wang (2011b). Genetic relatedness, $r_{XY}$ and the coancestry coefficient, $\theta_{XY}$ were then calculated as before.

Other estimators that were compared include those of Queller and Goodnight (1989), Wang (2002), Lynch and Ritland (1999), Lynch (1988), Ritland (1996), Wang (2007), and Milligan (2003), as implemented in the program COANCESTRY (Wang 2011a). Note that all the methods implemented in Wang (2011a) do not account for subpopulation structure (Table 2). However, all these methods account for multi-allelic data, which allow for equitable comparison with InRelate. The methods of Thornton *et al.* (2012) (REAP), Purcell *et al.* (2007) (PLINK), and Moltke and Albrechtsen (2013) (RelateAdmix), while more popular in recent years, are only applicable to di-allelic data (*e.g.*, SNP’s), and hence were not used for comparison in this manuscript.

**Table 2:** List of estimators tested and their references

	Label	Reference	Accounts for structure?
1	AW2007	Anderson and Weir 2007	Yes
2	Wang2011	Wang 2011b	Yes
3	MC2013_WI	Sethuraman 2013 with inbreeding	Yes
4	MC2013	Sethuraman 2013	Yes
5	TrioML	Wang 2007	No
6	Wang2002	Wang 2002	No
7	LynchLi	Lynch 1988, Li *et al.* 1993	No
8	LynchRi	Lynch and Ritland 1999	No
9	Ritland	Ritland 1996	No
10	QuellerG	Queller and Goodnight 1989	No
11	DyadML	Milligan 2003	No

### Bootstrapping and Pedigree Assignment

Under the assumption that sampled loci between two individuals *X* and *Y* are independent, we can obtain variance in estimation of relatedness by bootstrapping over loci. For every pair of individuals, loci are sampled with replacement to construct bootstrap replicates, and relatedness is estimated under the maximum likelihood framework. I then construct 95% confidence intervals of the estimated relatedness values. Simulated bootstrap standard errors are calculated as:

$$SE\left({\hat{\theta }}_{XY}\right)=\sqrt{\frac{\sum_{b=1}^{B}{\left({\hat{\theta }}_{XY,b}-{\bar{\hat{\theta }}}_{XY,b}\right)}^{2}}{B-1}}$$ (5)

where *B* is the number of bootstrap replicates and:

$${\bar{\hat{\theta }}}_{XY,b}=\frac{{\hat{\theta }}_{XY,b}}{B}$$ (6)

These variance estimates are then used in a series of Wald Tests, compared to a normal distribution, to assign relatedness categories to each pair of relatedness estimates. The Wald Test statistic is calculated as:

$$\frac{{\hat{\theta }}_{XY}-\theta_{0}}{SE\left({\hat{\theta }}_{XY}\right)}$$ (7)

After correcting for multiple testing by the Bonferroni method, pairs are assigned to a relatedness category at a p-value threshold of 0.05. Relatedness categories tested include: MonoZygotic twins - MZ, Full Siblings - FS, Half Siblings - HS, First Cousins - FC, Parent-Offspring - PO, Second Cousins - SC, AvunCular - AC, and UnRelated - UR.

### Simulations

Five separate sets of multi-allelic genomic data were simulated to test the performance of relatedness estimates using InRelate (MC2013, hereon), against other estimators. In all scenarios, subpopulations from which individuals were sampled from were assumed to be the ‘true’ subpopulation, for comparison with other methods. Admixture proportions and subpopulation allele frequencies for all analyses were obtained by performing runs of MULTICLUST (Sethuraman 2013). MULTICLUST uses an EM algorithm to estimate parameters under the admixture model (Pritchard *et al.* 2000b), and extends the method of Alexander *et al.* (2009) for multi-allelic data. It is much faster than STRUCTURE, and does not have MCMC convergence and mixing problems. Convergence of the EM algorithm was assumed if the log likelihood was not increasing by $\geq 10^{-6}$ in all scenarios.

#### Scenario 1: Hierarchical Island Model:

Under Scenario 1, all initial allele frequencies were simulated at 50 diploid, codominant, multi-allelic (maximum of 50 allelic variants per locus), unlinked loci, using Easypop v.1.7 (Balloux 2001). The Hierarchical Island Model was used, wherein each total population (out of 3) is comprised of subpopulations, which are in turn comprised of smaller subpopulations. I varied the number of subpopulations (*K*) to be one of 3, 5, 10, or 15. To allow for genetic admixture, I specified relatively greater levels of gene flow of 0.01 total proportion of migrant females and males per generation, between subpopulations inside each population, and relatively lower gene flow of 0.001 total proportion of migrant females and males per generation, between populations. Subpopulation sizes of 25 males and 25 females per subpopulation were held constant across generations. A forward-time simulation was performed for 3000 generations, and I utilized the last generation’s allele frequency distribution for all further simulations. All populations at generation 3000 were tested for Hardy-Weinberg Equilibrium (HWE).

#### Scenario 2: Island Model:

Under Scenario 2, I simulated multi-allelic genomic data using the same demographic parameters as in scenario 1 using a single Island Model (K=1), with no migration.

#### Simulating related dyads:

I then simulated k=1000 replicate dyads each of Parent-Offspring (PO), Full Siblings (FS), Half Siblings (HS), First Cousins (FC), and UnRelated (UR) individuals under different levels of known population subdivision (K=3,5,10,15). For FS dyads, two parents were randomly picked from the same subpopulation, and two offspring were created from their multilocus genotypes by randomly sampling their allele distribution from either parent. Since these loci are unlinked, I did not explicitly account for IBD tract length distribution. For HS dyads, one shared parent, and two other parents were simulated, and offspring generated from each cross. For FC dyads, a pair of FS dyads were created first, then their mates were randomly picked from the same subpopulation, to create offspring from each cross. PO dyads were picked similar to the FS simulation, with two parents being sampled randomly from the same subpopulation to create an offspring, and one of the parents were sampled as part of the dyad. UR dyads were created by randomly sampling two individuals from the same subpopulation.

Admixture proportions and subpopulation allele frequencies were estimated using MULTICLUST (Sethuraman 2013) at the ‘true’ assumed number of subpopulations (*i.e.*, K=1,3,...,15). These estimates were then used in determining pairwise genetic relatedness with InRelate.

I estimated $F_{st},$ using the *geneclust* package in R, and utilized those estimates in the same IBD-IBS framework in R to obtain pairwise relatedness by the methods of Anderson and Weir (2007) and Wang (2011b). The package geneclust implements the method of Weir and Cockerham (1984) to obtain a normalized multi-locus global *θ* estimate. For comparison with methods that did not account for population structure, I used the program COANCESTRY (Wang 2011a). Table 2 shows a summary of methods tested in this manuscript.

#### Scenario 3: Effect of number of loci:

To quantify the effect of increasing the number of genotyped loci on estimates of genetic relatedness, I simulated datasets under the same models specified in scenario 1 above, and the number of observed loci were varied between 10 and 40, to simulate a realistic scenario wherein individuals are genotyped at <50 variant STR loci.

#### Scenario 4: Effect of method of estimating $F_{st}$:

Under scenario 4, I was interested in how the estimation of $F_{st}$ affected estimates of genetic relatedness using the methods of Anderson and Weir (2007) and Wang (2011b), against MC2013 (InRelate using all 9 IBD states), and MC2013WI (InRelate using only the last 3 IBD states, assuming an outbred population, *sensu* Moltke and Albrechtsen 2013). To study this, I simulated a total of 1000 individuals distributed among K=3 subpopulations, genotyped at 50 STR loci (≤50 allelic states per locus), with a mutation rate of $1\times 10^{-6}$ mutations per generation, and a constant bidirectional migration rate of 0.001 of total individuals per generation, for 5000 generations. This gives a theoretical $F_{st}=\frac{1}{\left(1+4Nm\right)}$ of 0.2, while Weir and Cockerham’s normalized Θ estimated at generation 5000 by *geneclust* was 0.1038. Hundred FS pairs were simulated from the generation 5000 population as described above. Allele frequency distribution of the generation 5000 population was used in estimating relatedness by the methods of Anderson and Weir (2007) and Wang (2011b). Admixture proportions and subpopulation allele frequencies for use by MC2013 and MC2013WI estimators were obtained using MULTICLUST at K=3 as before. To compare the performance of the methods of Anderson and Weir (2007) and Wang (2011b), I estimated relatedness under both methods using (a) theoretical $F_{st}$ of 0.2, and (b) using the estimated Weir and Cockerham $\Theta_{st}$ of 0.1038.

#### Scenario 5: Effect of label-switching:

Under scenario 5, I was interested in understanding how ‘label-switching’ affected estimates of genetic relatedness in methods that accounted for population structure. ‘Label-switching’ in this context refers to misclassification of individuals to subpopulations. To study this, I used the same dataset simulated for scenario 3, switched the labels of either 0.1, 0.5 or 1.0 fraction of the total population, and re-estimated Weir and Cockerham’s $\Theta_{st}$ Weir and Cockerham (1984), and genetic relatedness using the methods of Anderson and Weir (2007) and Wang (2011b). Since population assignment is not *a priori* for the MC2013 and MC2013WI methods, I used the same results obtained from scenario 3 for comparison with the methods of AW2007 and Wang2011.

#### Error and Bias:

Deviation from true relatedness was examined by calculating the Mean Square Error (MSE). MSE is measured as:

$$\frac{1}{R}\sum_{i=1}^{R}{\left(\hat{r_{i}}-r_{true}\right)}^{2}$$ (8),

where *R* is the total number of replicate dyads (here 1000), $\hat{r_{i}}$ is the relatedness estimated using one of the above methods, and $r_{true}$ is the true relatedness value, $r_{xy},$ which is 0.5 for PO and FS dyads, 0.25 for HS dyads, 0.125 for FC dyads, and 0.0 for UR dyads. Bias was calculated as the deviation of the mean for all k=1000 replicates under each scenario from the true mean.

$${\bar{r}}_{true}-{\bar{\hat{r}}}_{i}$$ (9)

#### Scenario 6: Bootstrapping:

For bootstrap analyses, I simulated another dataset from the above data set of K=3 subpopulations, genotyped over 300 loci. I picked 5 dyads each of FS, HS, PO, FC, and UR individuals (total of 50 individuals). Boostrap datasets (200 replicates) were then simulated, with 50 individuals each by resampling loci with replacement. For each dataset, the true subpopulation structure was assumed to be comprised of K=3 subpopulations. Admxiture proportions and allele frequencies were computed using MULTICLUST (at K=3), and relatedness was then estimated using InRelate. Relatedness category assignment was then performed using the procedure described above.

#### Scenario 7: HGDP-CEPH Data:

Rosenberg (2006) and several allied publications (also see Ramachandran *et al.* 2005, Rosenberg *et al.* 2006) describe the use of subsets of ‘unrelated’ individuals from the HGDP-CEPH Human Genome Diversity Cell Line Panel (*H1048* Cann *et al.* 2002, Rosenberg *et al.* 2005). In these studies, relatedness was estimated between all pairs of individuals from within each sampled locale using RELPAIR (Boehnke and Cox 1997, Epstein *et al.* 2000), and several putatively related individuals from both within and across sampled locations were identified. For the purpose of this manuscript, I mined the original *H1048* dataset for individuals reportedly related from within the African continent. The African continent was represented in this data set by 115 individuals, classified as Bantu (South Africa), Bantu (Kenya), Mandenka, Yoruba, San, Mbuti Pygmy, or Biaka Pygmy, and were genotyped at a total of 783 microsatellite loci. Average differentiation, measured as Nei’s $G_{st}$ between these sampled locations was estimated as 0.1169, using the method of Nei and Chesser (1983), which indicates ‘moderate’ levels of differentiation (Wright 1950). I estimated population structure within these 115 individuals using MULTICLUST, at an *a priori* K=7. Admixture proportions and subpopulation allele frequencies were then obtained for the 24 relatedness dyads reported in Rosenberg *et al.* (2005), and I used these in estimating pairwise relatedness using InRelate. Allele frequencies were calculated assuming sampled locations as subpopulations, and used in estimating relatedness by the methods of Anderson and Weir (2007) and Wang (2011b) for comparison. Note that RELPAIR (Boehnke and Cox 1997) utilizes recombination information to obtain genetic relatedness, and is therefore very different from all the other methods compared in this manuscript. For the purpose of this comparison, I used RELPAIR estimates as the ‘truth’ to measure concordance with MC2013 and MC2013WI.

### Data Availability

All simulated data, and R scripts can be accessed at https://github.com/arunsethuraman/inrelate.

## Results

### Scenario 1: Hierarchical Island Model

**Figure 2:**
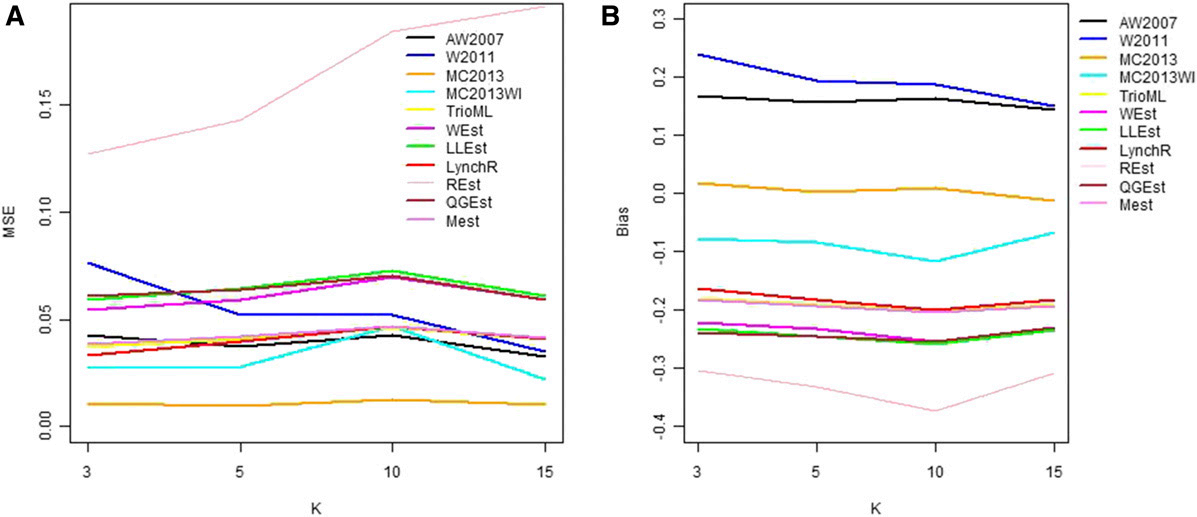
Comparing (a) MSE and (b) Bias in estimates of genetic relatedness between 1000 Full Sib (FS) dyads with increasing degree of subpopulation structure. Number of subpopulations (K) here was varied between K = 3 to K = 15 under the hierarchical island model described in Scenario 1.

**Figure 3:**
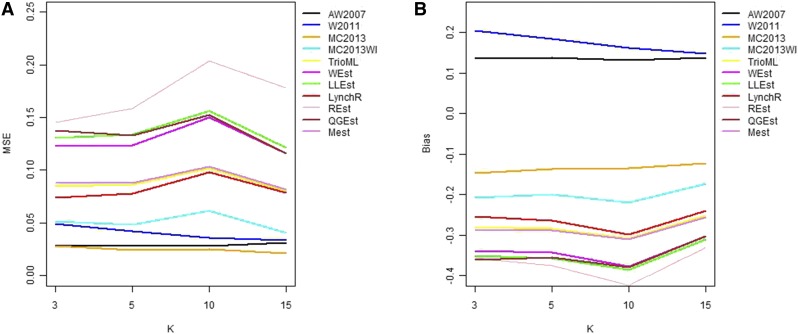
Comparing (a) MSE and (b) Bias in estimates of genetic relatedness between 1000 Half Sib (HS) dyads with increasing degree of subpopulation structure. Number of subpopulations (K) here was varied between K = 3 to K = 15 under the hierarchical island model described in Scenario 1.

**Figure 4:**
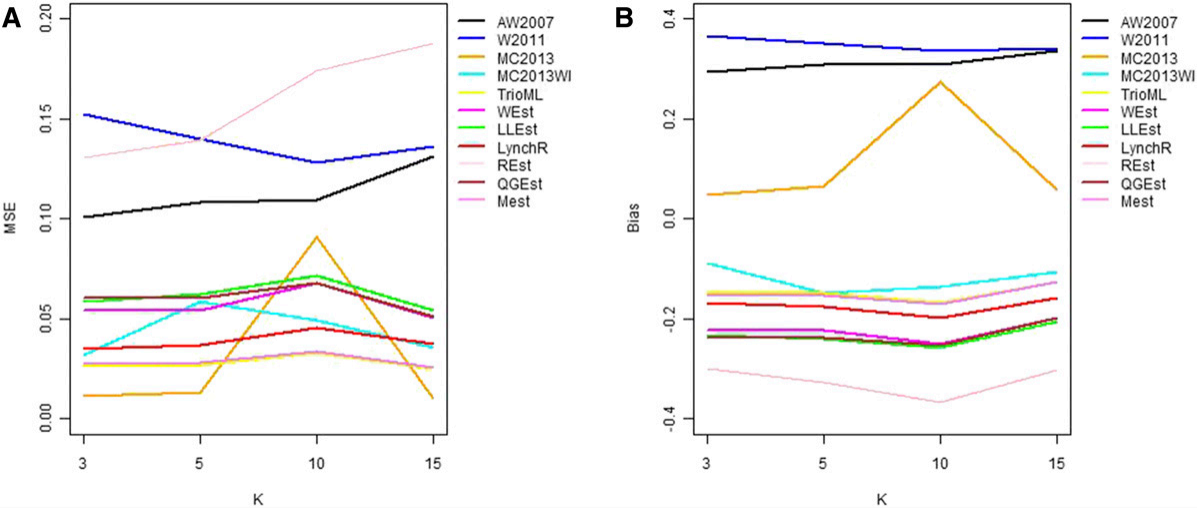
Comparing (a) MSE and (b) Bias in estimates of genetic relatedness between 1000 Parent Offspring (PO) dyads with increasing degree of subpopulation structure. Number of subpopulations (K) here was varied between K = 3 to K = 15 under the hierarchical island model described in Scenario 1.

**Figure 5:**
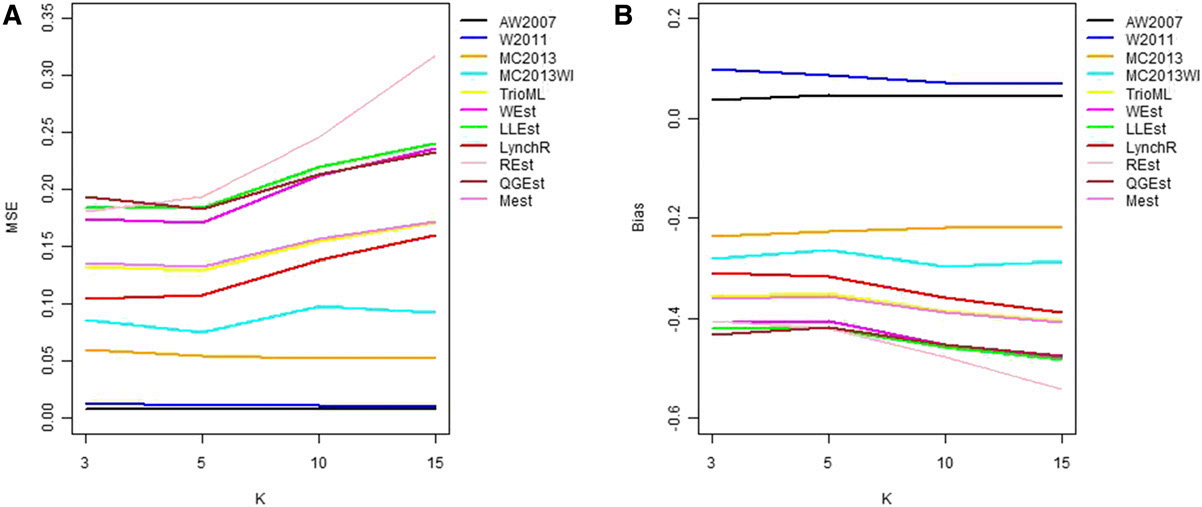
Comparing (a) MSE and (b) Bias in estimates of genetic relatedness between 1000 First Cousin (FC) dyads with increasing degree of subpopulation structure. Number of subpopulations (K) here was varied between K = 3 to K = 15 under the hierarchical island model described in Scenario 1.

**Figure 6:**
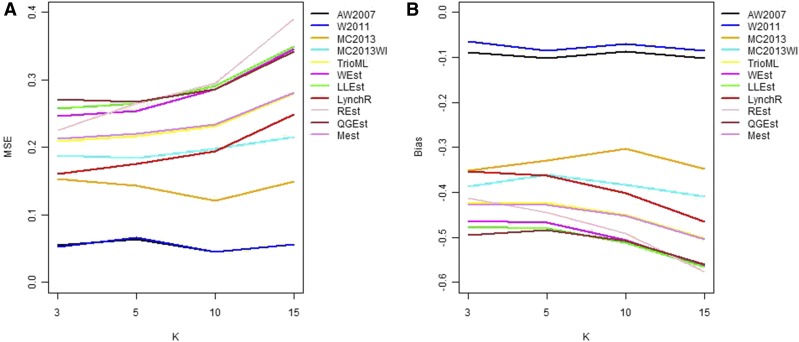
Comparing (a) MSE and (b) Bias in estimates of genetic relatedness between 1000 UnRelated (UR) dyads with increasing degree of subpopulation structure. Number of subpopulations (K) here was varied between K = 3 to K = 15 under the hierarchical island model described in Scenario 1.

**Figure 7:**
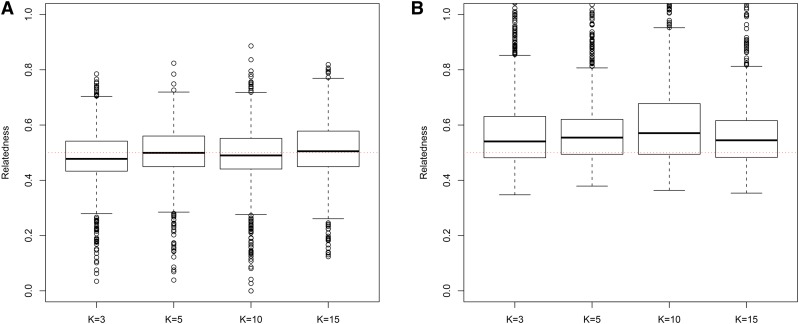
Distribution of estimates of genetic relatedness between 1000 Full Sib (FS) dyads with increasing degree of subpopulation structure using (a) MC2013, and (b) MC2013WI estimators implemented in InRelate. Number of subpopulations (K) here was varied between K = 3 to K = 15 under the hierarchical island model described in Scenario 1. True relatedness between full sibs = 0.5 is indicated using the dotted red line.

**Figure 8:**
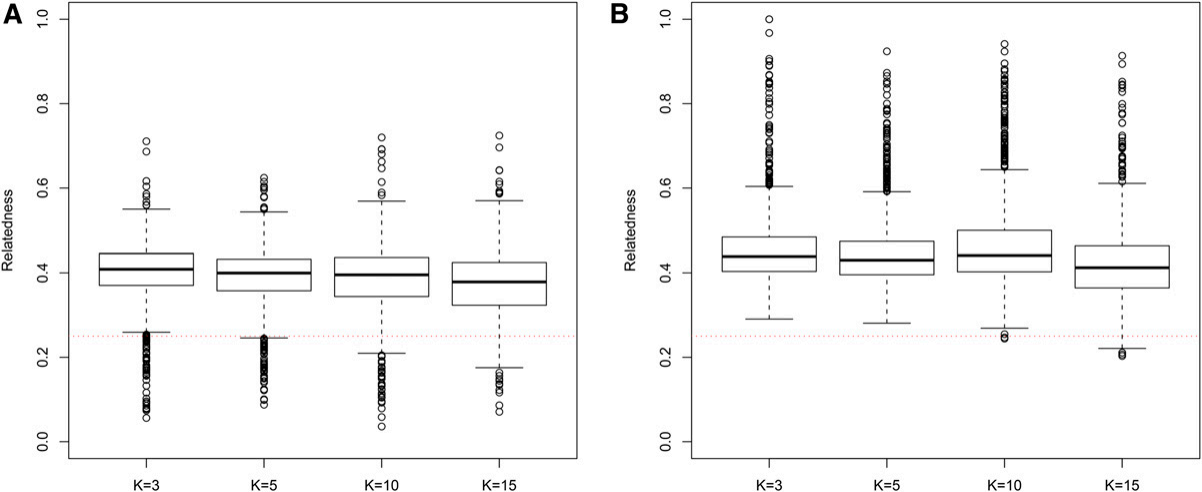
Distribution of estimates of genetic relatedness between 1000 Half Sib (HS) dyads with increasing degree of subpopulation structure using (a) MC2013, and (b) MC2013WI estimators implemented in InRelate. Number of subpopulations (K) here was varied between K = 3 to K = 15 under the hierarchical island model described in Scenario 1. True relatedness between half sibs = 0.25 is indicated using the dotted red line.

**Figure 9:**
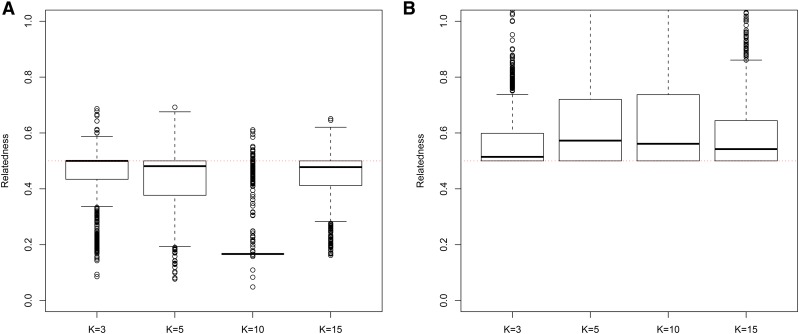
Distribution of estimates of genetic relatedness between 1000 Parent Offspring (PO) dyads with increasing degree of subpopulation structure using (a) MC2013, and (b) MC2013WI estimators implemented in InRelate. Number of subpopulations (K) here was varied between K = 3 to K = 15 under the hierarchical island model described in Scenario 1. True relatedness between parent-offsprings = 0.5 is indicated using the dotted red line.

**Figure 10:**
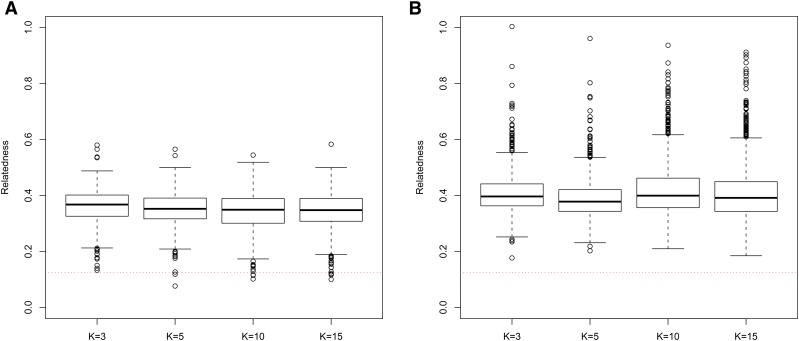
Distribution of estimates of genetic relatedness between 1000 First Cousin (FC) dyads with increasing degree of subpopulation structure using (a) MC2013, and (b) MC2013WI estimators implemented in InRelate. Number of subpopulations (K) here was varied between K = 3 to K = 15 under the hierarchical island model described in Scenario 1. True relatedness between first cousins = 0.125 is indicated using the dotted red line.

**Figure 11:**
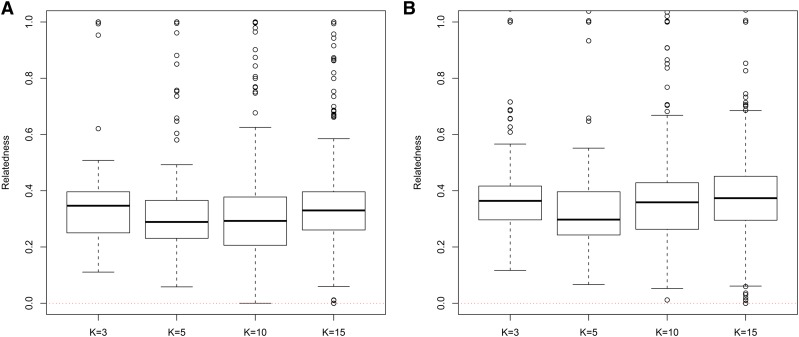
Distribution of estimates of genetic relatedness between 1000 UnRelated (UR) dyads with increasing degree of subpopulation structure using (a) MC2013, and (b) MC2013WI estimators implemented in InRelate. Number of subpopulations (K) here was varied between K = 3 to K = 15 under the hierarchical island model described in Scenario 1. True relatedness between half sibs = 0.0 is indicated using the dotted red line.

In general, in all scenarios that measured genetic relatedness among FS, PO, and HS dyads, the InRelate estimator (MC2013) performed better, or comparably with the AW2007 (Anderson and Weir 2007) and Wang2011 (Wang 2011b) estimators (Figures 2, 3, 4, 5, 6). FS and PO relatedness had the least bias, compared to all other estimators. Interestingly, MC2013 and MC2013WI underestimated relatedness in FC and UR dyads when compared to the AW2007 and Wang2011 estimators. Distributions of estimated relatedness using MC2013 and MC2013WI are shown in Figures 7, 8, 9, 10, and 11.

The other estimators that did not account for population structure consistently over-, or under-estimated genetic relatedness between dyads, with large mean squared errors (MSE). It was also noted (Wang 2011b) that all estimators that ignored population genetic structure had increasing bias, with an increase in the degree of population genetic structure, except in the inference of PO dyads, and UR dyads.

Correspondingly, MC2013 had the lowest MSE in the estimation of relatedness in FS, PO, and HS dyads, while the methods of AW2007 and Wang2011 had the lowest MSE for FC and UR dyads. The Ritland (Ritland 2005) estimator, and the methods of Anderson and Weir (2007) and Wang (2011b), had the highest MSE for PO dyads, while the Ritland estimator (Ritland 2005) had the highest MSE in all the cases. The estimators of Queller and Goodnight (1989) (QuellerG), Lynch and Ritland (1999) (LynchRi), and Wang (2007) (TrioML) performed similarly, with higher bias and MSE, than MC2013. Also, the estimators of Ritland (1996), Queller and Goodnight (1989) may have values <0 or >1, but these were not truncated to fall inside this range, as performed by Wang (2011b) in order to observe the true trend in estimation of relatedness.

### Scenario 2: Island Model

**Figure 12:**
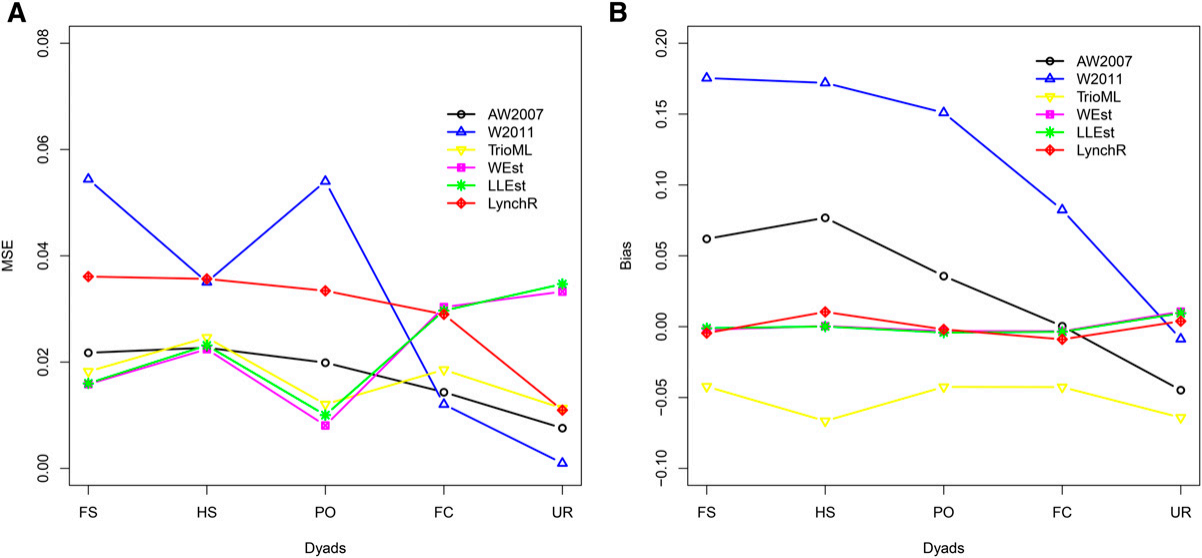
(a) MSE and (b) Bias in estimates of genetic relatedness between 1000 Full Sib (FS) dyads sampled from a panmictic population (K = 1) under Scenario 2, as described in the methods. Methods compared in this figure are those of Anderson and Weir (2007), Wang (2011b), Wang (2007), Wang (2002), Lynch (1988), and Lynch and Ritland (1999).

**Figure 13:**
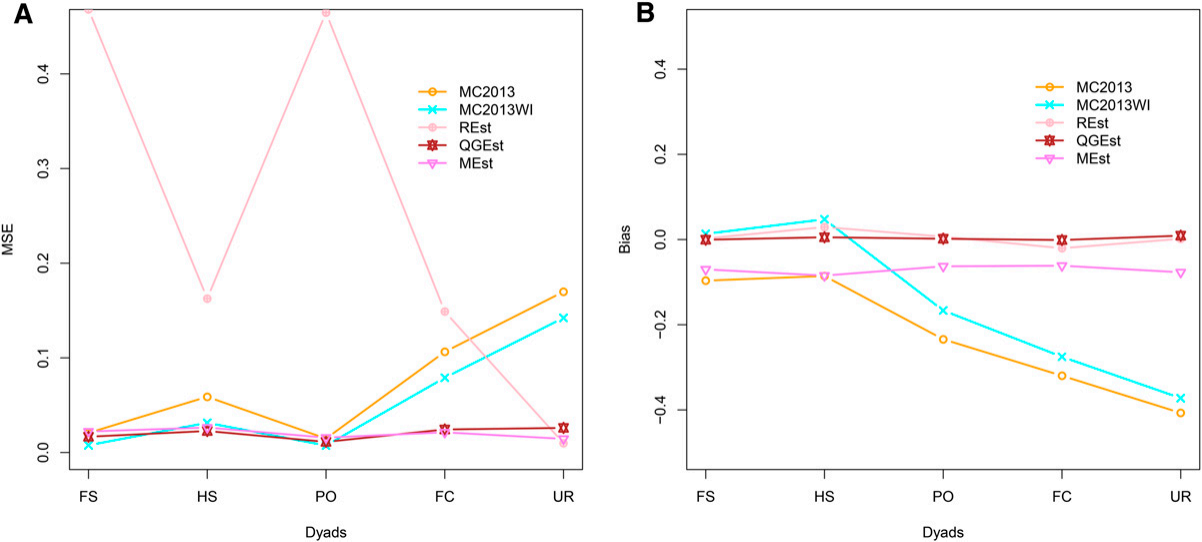
(a) MSE and (b) Bias in estimates of genetic relatedness between 1000 Full Sib (FS) dyads sampled from a panmictic population (K = 1) under Scenario 2, as described in the methods. Methods compared in this figure are MC2013, MC2013WI, Ritland (1996), Queller and Goodnight (1989), and Milligan (2003).

In the absence of population structure, under a panmictic island model, all methods performed comparably, with low MSE and bias for all FS, PO, HS dyads. The method of Ritland (1996) had considerably higher MSE compared to all the other methods in the estimation of FS, PO, HS and FC dyads. The MC2013 and MC2013WI estimators have higher MSE and bias in determining relatedness between FC and UR dyads (see Figures 12, 13).

### Scenario 3: Effect of Number of Loci

**Figure 14:**
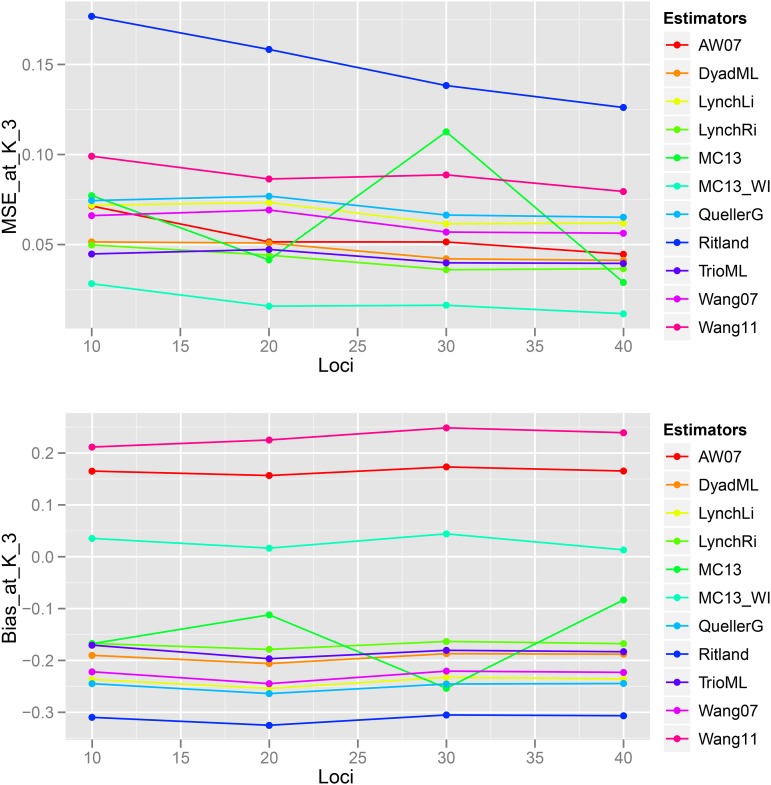
Bias and Mean Squared Error in estimates of genetic relatedness between 1000 Full Sib (FS) dyads sampled from K=3 subpopulations, simulated under Scenario 5, with increasing number of genotyped loci between L = 10 and L = 40.

**Figure 15:**
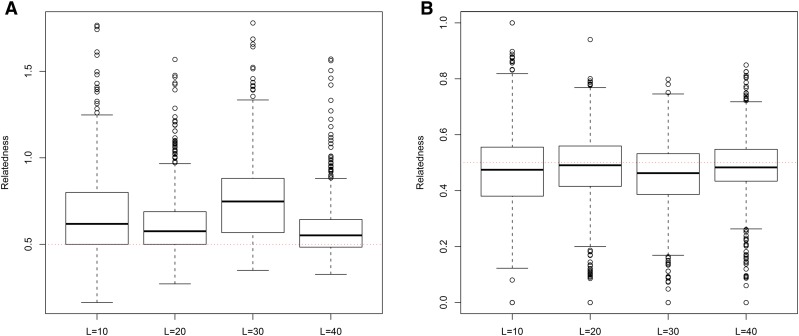
(a) MC2013 and (b) MC2013WI estimates of genetic relatedness between 1000 Full Sib (FS) dyads sampled under Scenario 5 (K = 3), by varying the number of loci sampled between L = 10 to L = 40. True estimate of relatedness between Full Siblings = 0.5 is shown in the dotted red line.

**Figure 16:**
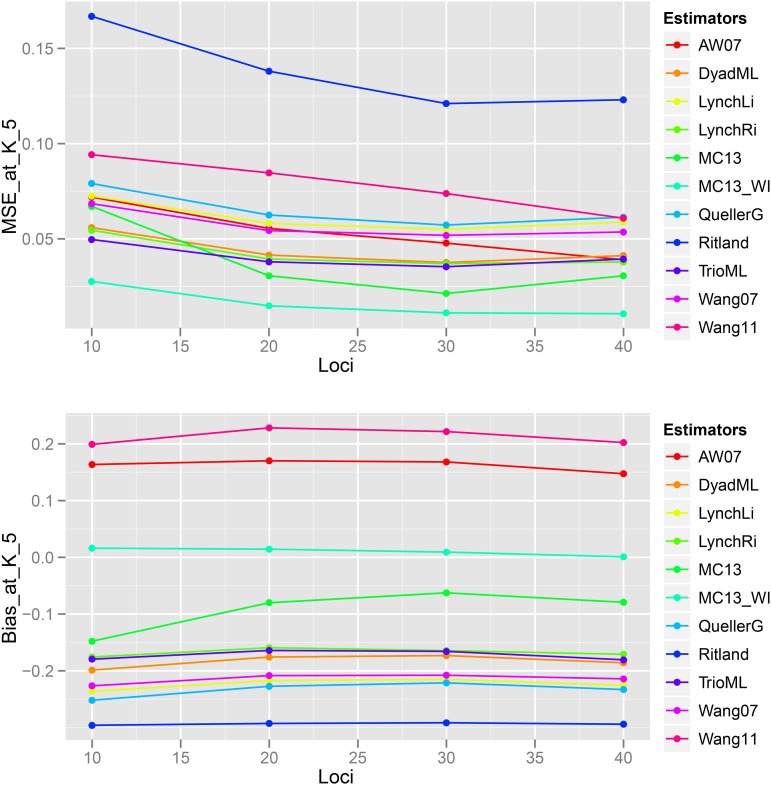
Bias and Mean Squared Error in estimates of genetic relatedness between 1000 Full Sib (FS) dyads sampled from K=5 subpopulations, simulated under Scenario 5, with increasing number of genotyped loci between L = 10 and L = 40.

**Figure 17:**
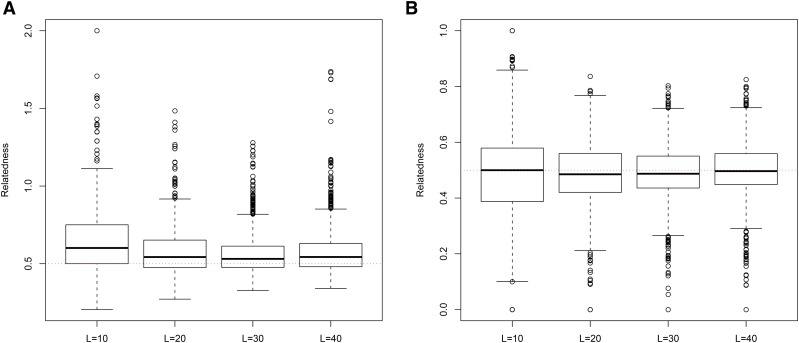
(a) MC2013 and (b) MC2013WI estimates of genetic relatedness between 1000 Full Sib (FS) dyads sampled under Scenario 5 (K = 5), by varying the number of loci sampled between L = 10 to L = 40. True estimate of relatedness between Full Siblings = 0.5 is shown in the dotted red line.

**Figure 18:**
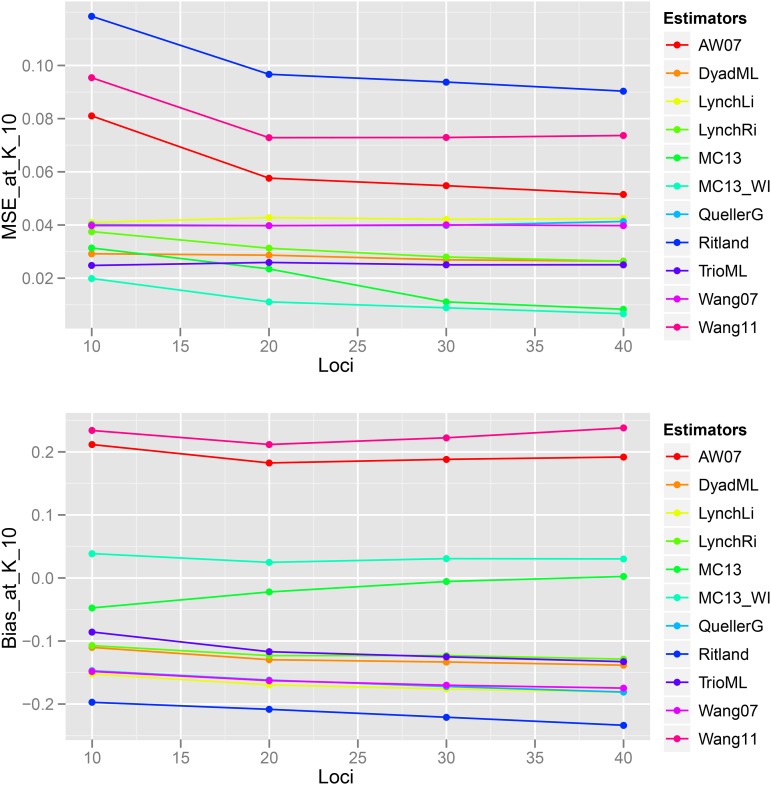
Bias and Mean Squared Error in estimates of genetic relatedness between 1000 Full Sib (FS) dyads sampled from K=10 subpopulations, simulated under Scenario 5, with increasing number of genotyped loci between L = 10 and L = 40.

**Figure 19:**
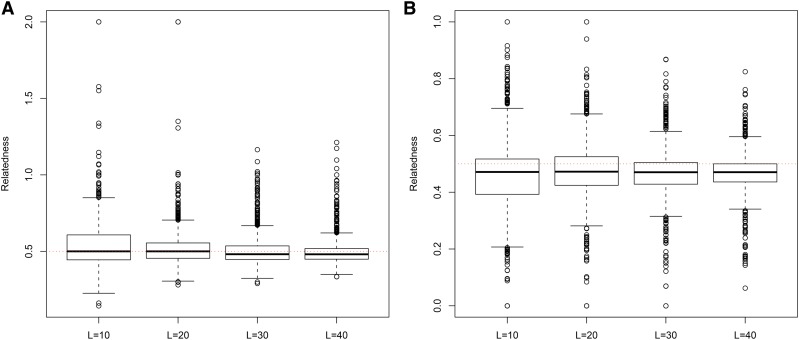
(a) MC2013 and (b) MC2013WI estimates of genetic relatedness between 1000 Full Sib (FS) dyads sampled under Scenario 5 (K = 10), by varying the number of loci sampled between L = 10 to L = 40. True estimate of relatedness between Full Siblings = 0.5 is shown in the dotted red line.

Bias and MSE estimates of pairwise genetic relatedness in FS dyads showed a trend of decrease with an increase in the number of loci (Figures 14, 15, 16, 17, 18, 19) across all estimators at K=3, 5, and 10, indicating the relative better estimation with increased genotypic information. Estimates of relatedness at K = 3, 5 and 15 are shown in Figures 15, 17, and 19 respectively. In general, InRelate had the least bias and least MSE in estimation of FS dyads across different levels of available information, measured as a function of the number of loci, with and without accounting for inbreeding (Figures 14, 16, 18). The estimator that accounted for inbreeding (MC2013WI) outperformed all other estimators with the least bias and MSE in estimation of FS relatedness. All other estimators of relatedness which did, or did not did not account for subpopulation structure performed with consistent decrease in bias and MSE with increase in the number of analyzed loci, as expected. The Ritland estimator was the least accurate, at K=3,5,10, across L=10,20,30,40, followed by the estimators of Anderson and Weir (2007), and Wang (2011b).

### Scenario 4: Effect of method of estimating $F_{st}$

**Figure 20:**
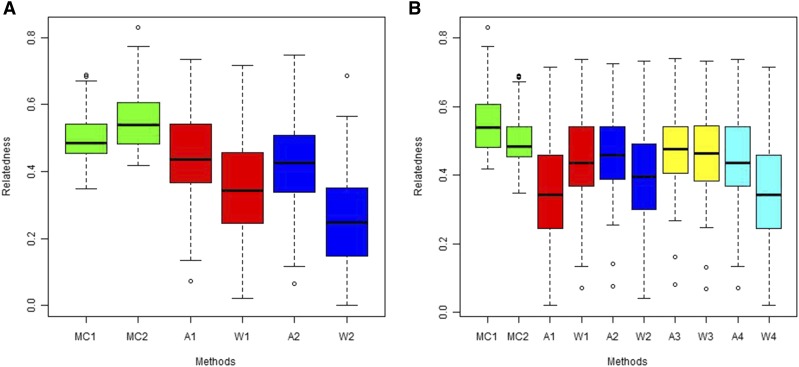
Estimates of relatedness for 1000 FS dyads simulated under the panmictic island model (K = 1). (a) Estimates of relatedness under Scenario 3 where method of estimating $F_{st}$ was varied. MC1 denotes the method of MC2013, MC2 is MC2013 accounting for inbreeding, A1 is the method of Anderson and Weir (2007) using the estimated $\Theta_{st}$ of Weir and Cockerham (1984), W1 is the method of Wang (2011b) using estimated Θ, A2 and W2 denote the above methods using expected $F_{st}.$ (b) Estimates of relatedness under Scenario 4, where the population ID’s were shuffled to simulate ‘label switching’. MC1, MC2, A1 and W1 are the same as before. A2 and W2 are the methods of Anderson and Weir (2007) and Wang (2011b) respectively, with 0.1 proportion of labels shuffled, A3 and W3 have 0.5 proportion of labels shuffled, and A4 and W4 have 1.0 proportion of labels shuffled.

The methods of Anderson and Weir (2007) and Wang (2011b) have larger confidence intervals in estimating relatedness in FS dyads, with the $\Theta_{st}$ of Weir and Cockerham (1984) having lower deviation from the truth ($r_{xy}=0.5$), compared to the theoretical $F_{st}.$ The MC2013 and MC2013WI methods outperform both methods with smaller confidence intervals around the mean (as shown in Figure 20).

### Scenario 5: Effect of ‘label-switching’

InRelate estimators do not have problems with ‘label-switching’, since population assignment is determined by the clustering method, and hence the ancestry proportions and allele frequencies are recomputed every time. On the other hand, both the methods of Anderson and Weir (2007) and Wang (2011b) show increased deviation from the mean (true $r_{xy}=0.5$) when labels are switched, due to the erroneous computation of differentiation (See Figure 20).

### Scenario 6: Bootstrapping

**Figure 21:**
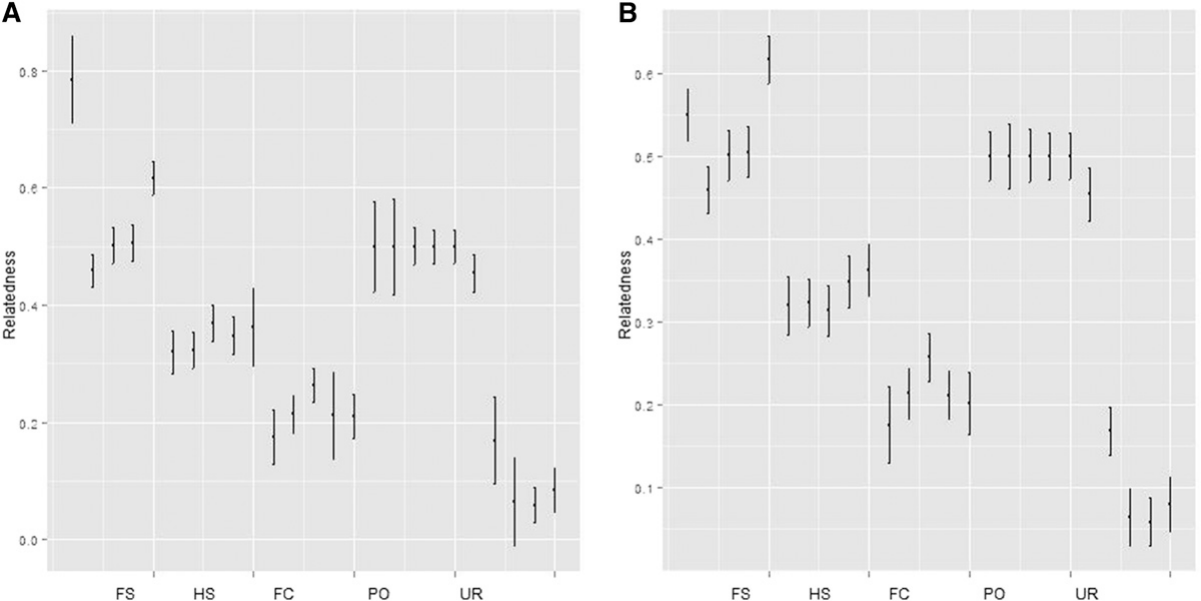
(a) MC2013 and (b) MC2013WI relatedness estimates and confidence intervals for 5 different relatedness categories, constructed using 200 bootstrap replicates under Scenario 6. The simulation used K = 3 subpopulations, and a total of 5 dyads of FS, HS, PO, FC, and UR individuals were picked.

Out of the 5 dyads of FS, HS, PO, FC and UR categories were correctly assigned after 200 bootstrap iterations in 44% of pairs. All Parent-Offspring pairs were correctly assigned, while two each of FS, FC, and UR pairs were correctly assigned. None of the HS pairs were significantly assigned to any category. Plots of confidence intervals around estimates using both the MC2013 and MC2013WI estimators are shown in Figure 21.

### Scenario 7: HGDP-CEPH Panel

**Figure 22:**
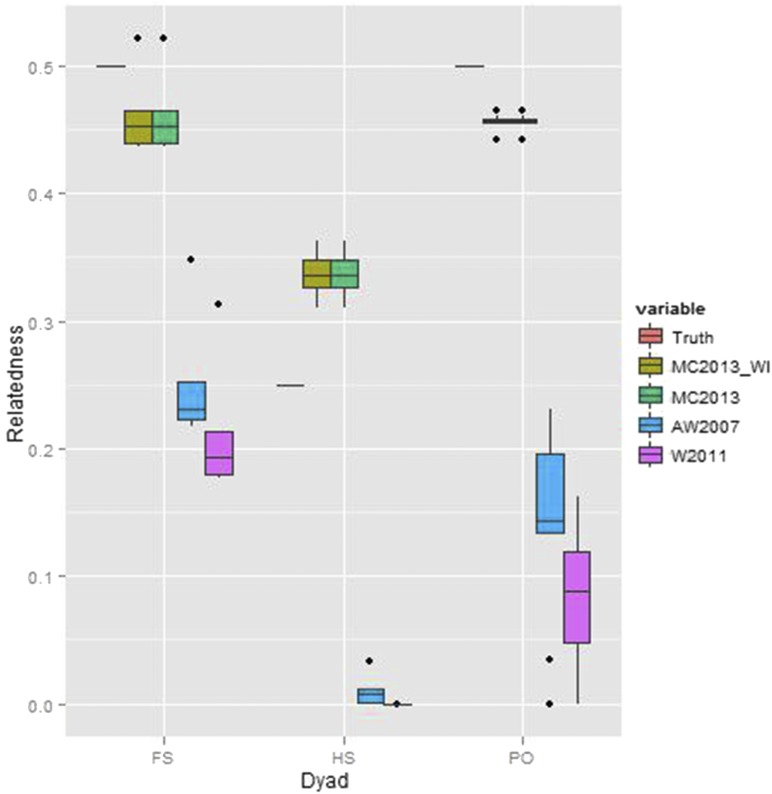
Relatedness estimates between 24 related dyads sampled from 6 locations in Africa, which were previously reported to be either FS, HS (or avuncular), or PO dyads by Rosenberg *et al.* (2005) using REAP (Thornton *et al.* 2012). The REAP estimates are plotted as the ‘True’ estimate, while other estimators compared are those of MC2013, MC2013WI, AW2007, and Wang2011.

Across 24 dyads which were either identified as FS, HS (or Avuncular), or PO by Rosenberg *et al.* (2005), the MC2013 and MC2013WI estimators outperformed the methods of Anderson and Weir (2007) and Wang (2011b) (see Figure 22), with consistently lower bias (MC2013WI - mean bias = 0.0114 (sd = 0.0667), MC2013 - mean bias = 0.0114 (sd = 0.0667), AW2007 - mean bias = -0.2857 (sd = 0.0779), Wang2011 - mean bias = -0.3204 (sd = 0.0895)). The MSE was also considerably lower (MC2013WI - 0.0044, MC2013 - 0.0044, AW2007 - 0.0874, Wang2011 - 0.1103) when comparing the MC2013 estimators with AW2007 and Wang2011. As reported before, these populations are ‘moderately’ differentiated (with a $G_{st}$ = 0.1169), and have historically been reported to have significant levels of gene flow or admixture, as well as exhibiting serial founder effects (see Tishkoff *et al.* 2009, Ramachandran *et al.* 2005).

## Discussion

The presence of ancestral subpopulation structure affects estimates of pairwise genetic relatedness between individuals from the same subpopulation, owing to pervasive inbreeding, and non-random mating in recent ancestral generations.

The primary goal of this paper was to develop a maximum-likelihood framework using an alternate parametrization, to estimate pairwise genetic relatedness between two individuals *X* and *Y*, while accounting for the ‘true’ genetic subpopulation structure in the population. This ‘true’ genetic subpopulation structure is unobserved and can be inferred from the data. Since the proposal of an admixture model by Pritchard *et al.* (2000b), several tools have been developed to estimate subpopulation structure, primarily to infer the number of subpopulations, *K*, admixture proportions (here $\eta_{ik}$), and subpopulation allele frequencies, $p_{kla}.$ These estimates have been applied widely, including to infer ancestral migration patterns (*e.g.*, Rosenberg *et al.* 2002,Eriksson and Manica 2012), in association studies (*e.g.*, Collins-Schramm *et al.* 2002), and to inform conservation decisions (see Allendorf *et al.* 2010). InRelate uses inferred information from population structure studies (using methods such as STRUCTURE (Pritchard *et al.* 2000b) or MULTICLUST (Sethuraman 2013) - see Liu *et al.* 2013) to inform the estimation of relatedness.

Across my simulations and analyses of the HGDP-CEPH African datasets, InRelate estimators of relatedness (MC2013 and MC2013WI) outperform several previously developed methods for relatedness estimation in admixed populations with considerably less error and bias. This accuracy is more pronounced particularly in between pairs of full siblings, parent-offspring, or half-siblings. The previously developed methods of Anderson and Weir (2007) and Wang (2011b) outperform InRelate in estimating first cousins or unrelated dyads in my simulations. As noted by Anderson and Weir (2007), estimates of relatedness in unrelated individuals are upwardly biased by all methods (see Figure 6)). I surmise this result is an artifact of ignoring subpopulation structure, in the presence of undetected ancient admixture, which results in an upward bias for all estimates. While MC2013 and MC2013WI account for this by using estimated subpopulation allele frequencies, the other estimators (AW2007(Anderson and Weir 2007, Wang2011(Wang 2011b) approximate it by using current allele frequencies, estimated from sampled populations.

Of note though are general difficulties in estimation of relatedness between first cousins, second cousins, and other more distantly related or unrelated pairs. These are also seen and reported by other likelihood methods (see Thompson 1975, Anderson and Weir 2007, Wang 2011b, Konovalov and Heg 2008), other estimators that use summary statistics (see Lynch and Ritland 1999, Blouin 2003, Anderson and Weir 2007, Wang 2002), and methods that utilize linkage or recombination information (see Pemberton *et al.* 2013, Rosenberg *et al.* 2005). This is primarily due to the fact that the most predominant relationship between two individuals is usually inferred, while the historical relatedness, due to evolutionary demographic processes, between them is ignored by most methods. Methods that account for this ‘deep’ relatedness are yet to be devised, and could help resolve issues with estimating deeper pedigrees, and relatedness between individuals. Wang (2011b) also notes this bias in estimating relatedness values close to the lower bound of 0 in the methods of Anderson and Weir (2007) and Wang (2011b).

Varying the number of loci minimally affects all relatedness estimators. This outcome may derive from variation in allele frequencies being sufficiently explained by the parameters of the admixture model (admixture proportions and subpopulation allele frequencies), as against biasing all estimates using a single non-varying parameter, *θ* (or $F_{st}$, *sensu* Anderson and Weir 2007 and Wang 2011b). Several methods can estimate this coefficient *θ* and each method has its own biases and efficiencies. This approach could potentially cause increased bias and MSE in using the estimators of Anderson and Weir (2007) and Wang (2011b), which could be addressed by utilizing a population structuring method to assign individuals to subpopulations, conditioning on that population structure in estimating *θ*. Regardless, increasing the number of sampled loci decreased bias of all estimators, as expected.

InRelate estimators do not have problems with ‘label-switching’, since the subpopulation structure is inferred from the data and not assumed *a priori* as in all other methods. Correspondingly, all allele frequencies, and ancestry proportions are re-calculated with switched labels, which are then used in estimates of relatedness. While all my analyses have inferred admixture proportions at the assumed ‘true’ subpopulation structure (*i.e.*, K), perhaps the true utility of this method would be if this K was inferred from the data, and the corresponding inferred admixture proportions and allele frequencies used in the estimation of relatedness. However, this is a statistical problem (Pritchard *et al.* 2000b, Falush *et al.* 2003, Hubisz *et al.* 2009, Sethuraman 2013, Alexander *et al.* 2009), with estimates of subpopulation allele frequencies and ancestry proportions confounded by (1) different demographic histories (Falush *et al.* 2016, (2) overparametrization, and a general improvement in the likelihood with increasing the parameter *K* (Evanno *et al.* 2005), and (3) issues with label switching (Jakobsson and Rosenberg 2007). InRelate and the method of Moltke and Albrechtsen (2013) are hence both affected by the ‘accuracy’ of estimates of structure and admixture parameters.

InRelate methods are of best utility when dealing with multi-allelic data, generated from individuals that are sampled from populations that are ancestrally structured, and generally outdo the methods of Anderson and Weir (2007), and Wang (2011b), which are both relatedness estimators under similar models. InRelate also does not require linkage maps, which makes it more utilitarian for estimating relatedness in non-model systems that don’t have detailed genomic information. I have also shown that InRelate outperforms all the methods implemented in the COANCESTRY (Wang 2011a) software, since all these methods do not account for ancestral population structure. However, the RelateAdmix method of Moltke and Albrechtsen (2013), which has been shown to outperform the methods of REAP (Thornton *et al.* 2012), PLink (Purcell *et al.* 2007), and KING (Manichaikul *et al.* 2010) is more applicable when analyzing SNP (di-allelic) data, generated from non-inbred populations that are recently admixed. When the underlying demographic history of the sampled individuals is unknown (or difficult to estimate), methods that are model-free, such as PC-Relate (Conomos *et al.* 2016) are bound to perform better (summarized in Ramstetter *et al.* 2017).
